# Is there a cognitive measure of neurodegeneration for amyloid-Aβ-ratio probable Alzheimer's disease patients?

**DOI:** 10.1177/13872877261444308

**Published:** 2026-04-21

**Authors:** Helmut Hildebrandt, Thomas Duning, Miriam Holland-Letz

**Affiliations:** 1 Department of Neurology, 62546 Klinikum Bremen-Ost, Bremen, Germany; 2 Department of Psychology, 11233 Carl von Ossietzky University Oldenburg, Oldenburg, Germany

**Keywords:** Alzheimer’s disease, course prediction, neurodegeneration, neuropsychological testing, total tau, Trail Making Test-B

## Abstract

**Background:**

Recent developments in the assessment of Alzheimer's disease (AD) have centered on differential diagnostic questions. Only a few studies have aimed to identify neuropsychological measures that allow the prediction of disease progression. However, this question is central to informing patients about their diagnosis and to decisions regarding the urgency and timing of treatment escalation.

**Objective:**

We analyzed which, if any, neuropsychological test results reflect the extent of neurodegenerative progression.

**Methods:**

This retrospective analysis included 290 patients divided into an Aβ-ratio + group (n = 146; AD biomarker profile) and an Aβ-ratio– group (n = 144; non-AD biomarker profile). The Aβ-ratio + group was further divided into four t-tau quartiles. The Aβ-ratio– group was subdivided into patients with normal (n = 94) or elevated t-tau (n = 50).

**Results:**

Regression and variance analyses demonstrated a correlation between Trail Making Test B (TMT-B) performance and t-tau levels in patients with an Aβ-ratio+, driven by differences between low and high tau values, but not in Aβ-ratio– patients. Several additional statistical control analyses endorsed this finding.

**Conclusions:**

We conclude that, for Aβ-ratio + patients, TMT-B performance may serve as a clinically accessible indicator of tau-related disease activity and the extent of neurodegeneration and may help identify patients at risk of faster progression if replicated in longitudinal studies.

## Introduction

During the last two decades, the diagnosis of Alzheimer's disease (AD) has seen several breakthrough findings. It is now widely recognized that AD is characterized by the accumulation of extracellular amyloid-β (Aβ) plaques and intracellular neurofibrillary tangles composed of hyperphosphorylated tau protein. According to the prevailing cascade model, amyloid deposition represents an early event, often preceding clinical symptoms by years or even decades, whereas tau pathology correlates more closely with neuronal loss and clinical severity.^1–4^ Hence, tau and Aβ alterations reflect different phases of a common disease process. Furthermore, neuropathological studies have shown that tau aggregation initially emerges in the transentorhinal and entorhinal cortex and hippocampus before spreading from medial temporal lobe structures to associative neocortical regions. This spread of tau pathology is thought to underlie the progressive involvement of broader cognitive domains beyond memory.^5–7^

These insights have led to a growing focus on biomarker-based diagnosis and substantial technical advances. Starting with the development of Aβ- and tau-specific PET tracers, it became possible for the first time to visualize the pathological hallmarks of AD. Subsequent advances included the recognition that the Aβ_42/40_ ratio, rather than the absolute Aβ levels alone, provides a more specific diagnostic indicator. Most recently, the development of blood-based parameters has opened the possibility of reducing the need for invasive cerebrospinal fluid (CSF) punctures. Consequently, the diagnosis of AD today is no longer based solely on clinical findings but is complemented by more objective results.^3,4^

In clinical routine practice, however, the diagnosis of AD is only half the way. While identifying AD is one step, understanding and communicating its course and progression is another. For instance, a sound prognosis is critical in guiding treatment planning, such as initiating passive immunization, particularly in cases where such interventions are not yet established for a large number of patients. Additionally, patients and their relatives need a clear framework for anticipating the progression of cognitive and functional decline as well as the associated implications for everyday life. However, knowledge about the probable course of AD still lags behind advances in diagnostic accuracy.

Several approaches to predict the course of AD have been made. Biomarkers such as neurofilament light chain and glial fibrillary acidic protein have been explored as an indicator of disease progression depending on the clinical stage (very mildly impaired or non-impaired, mild cognitive impairment (MCI) or dementia).^8,9^ Yet, their prognostic value remains limited. The same holds for MRI-based measures such as hippocampal atrophy. Although hippocampal atrophy correlates with memory functions, most studies have shown that atrophy alone is not a reliable predictor of future course.^5^

A more promising approach has emerged from the examination of total tau (t-tau) levels and cognitive status. T-tau represents a relatively nonspecific marker of neuronal loss. Several studies have demonstrated that elevated levels increase the likelihood of conversion from MCI to dementia.^10^ In addition, other studies that used PET tracers for tau have shown that there is a correlation between the accumulation of tau in the entorhinal and hippocampal cortex, memory function, and disease progression.^11,12^ Similarly, neuropsychological testing has shown that the conversional risk from MCI to dementia is higher than that of cognitively very mild or unimpaired patients, and once the decline begins, the rate of progression typically accelerates. Some studies have shown that neuropsychological test performance can start to decline up to 10 years before clinical diagnosis, even preceding measurable changes in amyloid status.^13^ Arguably, some of the neuropsychological test findings may also reflect risk of disease progression and t-tau levels.

Indeed, previous studies have provided evidence for correlations between various neuropsychological tests and t-tau levels.^14–16^ This suggests that both t-tau and performance on these cognitive measures reflect a common underlying process of neurodegeneration. For instance, Lin et al. (2009) reported a correlation between CSF t-tau levels and short-term memory performance on the Cognitive Abilities Screening Instrument in patients with AD, demonstrating that higher t-tau levels were associated with more severe impairment on this measure. Other neuropsychological tests have been found to correlate more closely with AD-specific Aβ status.^15,17–19^ However, while these tests show impaired performance very early in the disease process, they do not appear to correlate with disease progression. In other words, although such measures have proven to be valuable for the *detection* of Alzheimer's pathology, they are less informative regarding the *prediction* of neurodegenerative progression.

Vromen et al.^20,21^ investigated neurodegenerative progression in a longitudinal design. They demonstrated that cognitive decline among biomarker-defined AD patients differs depending on tau status and is associated with distinct biological pathways. Compared with amyloid-positive but tau-negative individuals, tau-related decline was linked to synaptic processes and faster cognitive deterioration. Although these findings help explain differences in the rate and mechanisms of decline, they do not clarify which specific cognitive functions are affected. In that study, cognitive change was assessed using a global measure (Mini-Mental Status Examination, MMSE). While suitable for detecting overall cognitive worsening, such a measure does not directly reflect the neurobiological stage of disease progression (e.g., cortical spread of pathology) and cannot differentiate domain-specific cognitive changes. Consequently, it has limited sensitivity for detecting variability in progression within a clinical stage, such as differences among individuals with MCI.^20,21^ Reijs et al.^18^ likewise examined neurodegenerative progression by investigating associations between CSF biomarkers and multiple memory domains across individuals with normal cognition, MCI, and dementia. Although CSF t-tau was associated with decline in episodic verbal and semantic memory, associations varied and were partly limited by interindividual heterogeneity. Moreover, this study focused on memory functions, which are typically affected early in the disease course, whereas other cognitive domains were not systematically examined.^18^ Thus, while prior work has begun to focus on markers of disease progression, it remains unclear which neuropsychological tests best track the degree of neurodegeneration and sensitively capture progression within a clinical stage (e.g., MCI).

In conclusion, neuropsychological tests may contribute to both early diagnosis of AD (comparable to AT in the AT/N model^4^) and prediction of disease activity in AD (comparable to the N in the AT/N model). From a theoretical perspective, the first group of tests is expected to capture region-specific and temporally sensitive changes in neural integrity, seen during the earlier phases of the AD course (Braak stage I to III).^1,2,17,22,23^ The second class, in contrast, likely measures more composite cognitive functions that are not specific to AD pathology. Performance on these tests should deteriorate more slowly and parallel to the spread of tangles. To date, this latter perspective has received comparatively limited systematic investigation.

We acknowledge that answering the question of prediction ultimately requires longitudinal studies. The present study aims to combine cross-sectional data on t-tau and neuropsychological test results to provide guidance on which tests may be particularly sensitive to the degree of tau-related neurodegeneration and may be used in future longitudinal research.

For this purpose, the following questions were examined:

1. Which neuropsychological test scores predict t-tau levels in AD patients compared to non-AD patients, as determined by linear regression analysis?
2. When dividing the group of AD patients into subgroups with different t-tau levels, is the relationship between t-tau and test performance linear or non-linear?
3. As a control:
  - : Does the global disease severity (very mild or non-impaired, MCI, demented), as defined by MMSE scores, account for the relation between neuropsychological test performance and t-tau levels, or is this association independent of the global cognitive status?
  - : Is the relation between these neuropsychological tests and t-tau AD specific or also present in other neurodegenerative disorders?

To answer these questions, we analyzed three groups: (1) 146 patients with Aβ-ratio+, (2) 94 patients with Aβ-ratio- and t-tau-, and (3) 50 patients with Aβ-ratio- and t-tau + level.

## Methods

### Patients

We collected data from 290 patients admitted to the neurological department of hospital Bremen-Ost over the last 5 years (2019 to 2024) and suffering from objective or subjective cognitive decline. Patients younger than 52 years old, with definite non-degenerative causes of their impairment (for example stroke or encephalitis) or with a t-tau score beyond 1900 pg/ml were excluded.

The patients were divided into two groups: those with a reduced Aβ_42/40_ ratio (Aβ-ratio + patients) and those without. The cut-off point of 0.068 was defined for the Fujirebio LUMIPULSE method, which was used by our laboratory. We used the Aβ-ratio results alone to categorize patients as probable or improbable AD cases, as phospho-tau is highly correlated with t-tau. Therefore, integrating phospho-tau scores into the patient classification could bias the t-tau score, which served as a marker for neurodegeneration.

Patients with a score higher than 0.068 were categorized as Aβ-ratio patients. This group was further subclassified as impaired or non-impaired using 405 pg/ml t-tau as the criterion, as proposed by our laboratory. Within the Aβ-ratio– group, 46% of patients were clinically diagnosed with MCI or presented with impairment in a single cognitive domain without meeting criteria for a specific clinical diagnosis. Depression and normal pressure hydrocephalus each accounted for 12% of cases. Parkinson's disease and vascular lesions were present in 6% of patients, respectively. A further 6% were classified as possible AD based on reduced absolute Aβ_1–42_ levels despite an Aβ_42/40_ ratio above the pathological cut-off. The remaining 1% comprised other diagnoses.

The retrospective re-evaluation project was approved by the ethics committee of the Medical Board of Bremen (approval number 905/2024) and was performed in accordance with the ethical standards set out in the Declaration of Helsinki.

### Neurological examination

The standard diagnostic examination protocol included medical history assessment, physical and neurological examination, laboratory testing, brain imaging and a CSF analysis. The extensive blood sample analysis included blood count, erythrocyte sedimentation rate, electrolytes (sodium, potassium, chloride), creatine, urea, transaminases, blood glucose, TSH, C-reactive protein, vitamin B 12, folic acid. Optional further blood analyses included b-vitamins, TPPA, immunological parameter, HIV, and copper metabolism. CSF samples were analyzed for cell count, total protein, lactate, glucose, IgG, IgA, IgM, borrelioses antibodies, Aβ_42_, and t-tau. CSF t-tau and Aβ_42/40_ were determined quantitatively using Fujirebio LUMIPULSE G assays, per manufacturer's instructions. All analyses were performed commercial at the LADR GmbH MVZ Bremen.

### Neuropsychological examination

All patients underwent a neuropsychological examination, including the German version of the CERAD-NAB.^24–26^ The German version of the CERAD-NAB is the consented assessment tool for German-speaking memory units and includes the MMSE, a short version of the Boston Naming Test, semantic and phonological (first-letter) fluency, figure copy and delayed figure copy recall, wordlist learning and wordlist delayed free recall, as well as recognition. Based on the results of a large (more than 1000 participants) control group, z-scores are offered, corrected for age (starting with 51 years), education, and gender.

In addition, the Digit Span Forward and -Backward test from the German version of the Wechsler Memory Scale^27^ was conducted, two experimental memory and one language comprehension test and the Beck's Depression Inventory^28^ was used to investigate mood and depression. However, for this analysis we focused on the CERAD-NAB results, because of their z-scores controlling for age, gender, and education.

The MMSE^29^ as part of the CERAD-NAB was used to grade the severity of the cognitive decline.

### Statistical analysis

We used JASP (Version 0.95.1) for all statistical analysis. Step wise linear regression analysis was used to analyze the prediction of t-tau scores by CERAD-NAB neuropsychological z-scores. Group comparisons were based on ANCOVA for Aβ-ratio + t-tau quartile and for Aβ-ratio- t-tau groups with MMSE and liquor/serum Albumin quotient as covariates.

## Results

### Patients

A total of 290 patients with subjective or objective cognitive decline were included in the analysis. There were 146 patients with an AD-typical biomarker profile (Aβ-ratio + patients) and 144 patients with a non-AD biomarker profile (Aβ-ratio- patients).

#### Aβ-ratio + group

For the purpose of the study, the Aβ-ratio + group was divided into four subgroups based on t-tau scores. Table 1 shows the size of the resulting (sub)groups and their clinical characteristics. Patients in the Aβ-ratio + group had a mean age of 73.5 years and a mean MMSE score of 23.5, indicating overall cognitive impairment in the range of mild cognitive impairment to mild dementia. The t-tau quartile groups scored similarly on the MMSE, even though the scores ranged from 17 to 30. There was an insignificant difference of one point between the first (mean MMSE = 23.9) and fourth quartile (mean MMSE = 22.9) groups. Thus, the groups did not differ in overall cognitive severity.

**Table 1.** Descriptive Statistics of Aβ-ratio + patients.

t-tau (pg/ml)	**Whole group**		**≤402**		**403–545**		**546–786**		**≥787**	
N	146		37		37		36		36	
	**Mean**	**SD**	**Mean**	**SD**	**Mean**	**SD**	**Mean**	**SD**	**Mean**	**SD**
Age (years)	73.5	7.2	73.9	7.2	75.6	7.1	72.8	6.1	71.8	8.0
Education (years)	13.6	2.5	13.8	2.3	13.0	2.7	13.8	2.5	13.9	2.4
MMSE	23.5	3.2	23.9	2.9	23.6	3.1	23.5	2.8	22.9	3.8
t-tau	616.8	319.9	307.6	70.1	472.3	45.5	625.7	54.1	1074.1	274.8
Aβ-ratio *	0.044	0.010	0.047	0.009	0.045	0.010	0.045	0.010	0.039	0.008
LS Alb. Quotient	6.77	2.76	6.91	2.76	6.99	2.62	6.54	2.85	6.63	2.91

*p = 0.001.

N: number of patients per group; MMSE: Mini-Mental State Examination; T-tau: total tau; LS Alb: Liquor/Serum Albumin.

The t-tau quartile subgroups did not differ significantly in any other characteristic (age, education, albumin quotient) except for the Aβ-ratio level. A Bonferroni-corrected t-test revealed that the group with the highest t-tau level differed significantly from each of the other groups (p < 0.05). However, comparison of the Aβ-ratio levels of the other groups revealed no significant differences.

#### Aβ-ratio- patients

The group of Aβ-ratio- patients was also divided into two subgroups: those without increased t-tau (<405 pg/ml, cut-off score defined by our laboratory) (Aβ-ratio- Tot Tau- patients) and those with increased t-tau (≥ 405 pg/ml) (Aβ-ratio- Tot Tau + patients). Table 2 informs about the clinical characteristics of these groups. Patients in the Aβ-ratio– group had a mean age of 69.4 years and a mean MMSE score of 24.9, consistent with mild cognitive impairment. Patients with and without elevated t-tau levels did not differ significantly in age, education, Aβ-ratio, or albumin quotient.

**Table 2.** Descriptive statistics of Aβ-ratio- patients.

t-tau (pg/ml)	**Whole group**		**≥405**		**< 405**	
N	144		50		94	
	**Mean**	**SD**	**Mean**	**SD**	**Mean**	**SD**
Age (in years)	69.4	8.8	70.0	7.9	69.1	9.3
Education (in years)	13.3	2.6	13.9	2.4	13.0	2.7
MMSE	24.9	3.8	24.8	3.7	25.0	3.9
t-tau	409.76	306.1	710.8	342.4	249.5	87.3
Aβ-ratio *	0.432	0.502	0.438	0.353	0.428	0.567
LS Albumin Quotient	7.50	3.74	7.57	3.22	7.46	4.00

No significant differences between the groups.

N: number of patients per group; MMSE: Mini-Mental State Examination; T-tau: Total Tau; LS Alb.: Liquor/Serum Albumin.

### Neuropsychological testing for Aβ-ratio + patients

#### Total tau and neuropsychological tests results

A stepwise linear regression analysis was performed in Aβ-ratio + patients with t-tau as dependent and CERAD neuropsychological test results (Boston Naming Test, Semantic word fluency, Phonematic word fluency, Wordlist learning total, Word list learning savings, Figure copy, Figure copy savings, TMT A + B) as independent variables, using p values < 0.05 as entry and above 0.1 for removal. Because the neuropsychological tests were standardized for age, education, and gender effects, these variables were not included in the analysis. The regression analysis was significant (p = 0.003) with an adjusted R^2^ of 0.064. The only test result which remained in the model was the TMT-B z-score (standardized beta: −0.269, p = 0.003). The condition index of the final model was 3.535, arguing against collinearity issues. Inspection of residuals versus fitted plot and residuals versus predicted argued for homogeneity of variance.

#### Three additional analyses to test the specificity of the findings

1. Controlling for Aβ-Ratio: Because of the significant difference in the Aβ-ratio (Table 1), we repeated the regression analysis by first entering the Aβ-ratio, then the different neuropsychological test results, and allowing for an interaction with TMT-B. This was done to exclude the possibility that the Aβ-ratio alone might explain the tau level. The regression analysis was significant (p < 0.001) with an overall adjusted R^2^ of 0.125 and revealed a significant effect of the Aβ-ratio (standardized beta: −0.267, p = 0.003), a significant effect of TMT-B with a standardized beta of −0.243 (p = 0.006), but no significant interaction between both. Inspection of residuals versus fitted plot and residuals versus predicted again argued for homogeneity of variance.
2. Controlling for Global Cognitive Status and Blood–Brain Barrier Integrity: to analyze whether the relation between t-tau and TMT-B performance is linear and to control for MMSE state and blood-brain barrier (BBB) permeability, we divided the Aβ-ratio + group into t-tau quartiles, and calculated a ANCOVA for TMT-B and t-tau quartiles groups controlling for MMSE (to exclude that the result could be explained by global deterioration) and the level of liquor/serum Albumin quotient (to control for vascular or neuroinflammatory risks factors). There was a significant main effect of group [F(3141): 2.906, p = 0.038] and a significant effect for MMSE [F(1141)20.456, p < 0.001] but no significant effect for the albumin quotient. Post hoc testing with Bonferroni correction revealed that the group with lowest t-tau score differed from the group with the highest t-tau (T: 2.944, p = 0.023).

This suggests that performance in the TMT-B reflects the level of t-tau, and therefore loss of neuronal integrity, even when the MMSE score and neuroinflammation are taken into account. Conversely, the significant MMSE score suggests that cognitive state plays an independent role in TMT-B performance.

We repeated this analysis using Aβ ratio + t-tau quartile groups and tested whether there were differences according to the albumin ratio, but there were none.

1. Specificity Analysis in Aβ-Ratio– Patients: In the next step, we analyzed if the relation between t-tau and TMT-B was specific for Aβ-ratio + patients or is more globally related to t-tau increase due to other brain diseases. For that reason, we used the Aβ-ratio- group (Table 2) and replicated the same stepwise linear regression analysis. The analysis yielded again significant results. However, in this case only WL-total entered the regression analysis (p < 0.001), explaining adjusted 0.101 R^2^ change.

We also repeated the ANCOVA for TMT-B performance by splitting the Aβ-ratio- group along with the cut-off score of 405 pg/ml t-tau into four quartile groups. This analysis revealed no significant results for the Aβ-ratio- t-tau groups, no significant effect for the Albumin quotient but a significant main effect for MMSE [F(1141): 32.836, p < 0.01].

## Discussion

This study found that in Aβ+ AD individuals, higher t-tau levels were significantly associated with poorer performance on the TMT-B. Importantly, this association could not be found in Aβ– individuals (i.e., non-AD biomarker profile), where t-tau levels were instead related to verbal learning performance.

One possible explanation for this specific relationship is that in the Aβ+ population, the combination of high t-tau levels and impaired TMT-B performance may reflect the stage of AD disease progression. Specifically, performance on the TMT-B requires a combination of working memory functions, visual search, and interference control, activating a bilateral frontoparietal network.^28,29^ These regions are not primarily affected in early Braak stages (I–III) used to classify AD progression, but become involved once tau spreads beyond the medial temporal lobe (Braak stage IV). Consequently, impaired TMT-B performance may represent a functional marker of more advanced or aggressive tau pathology. In contrast, in Aβ– individuals, t-tau levels were unrelated to TMT-B performance and instead linked to verbal learning deficits. This suggests that tau-related neuronal injury in Aβ– patients affects different neural systems, supporting the view that the TMT-B/t-tau association is specific to AD-typical pathology.

**Figure 1.**
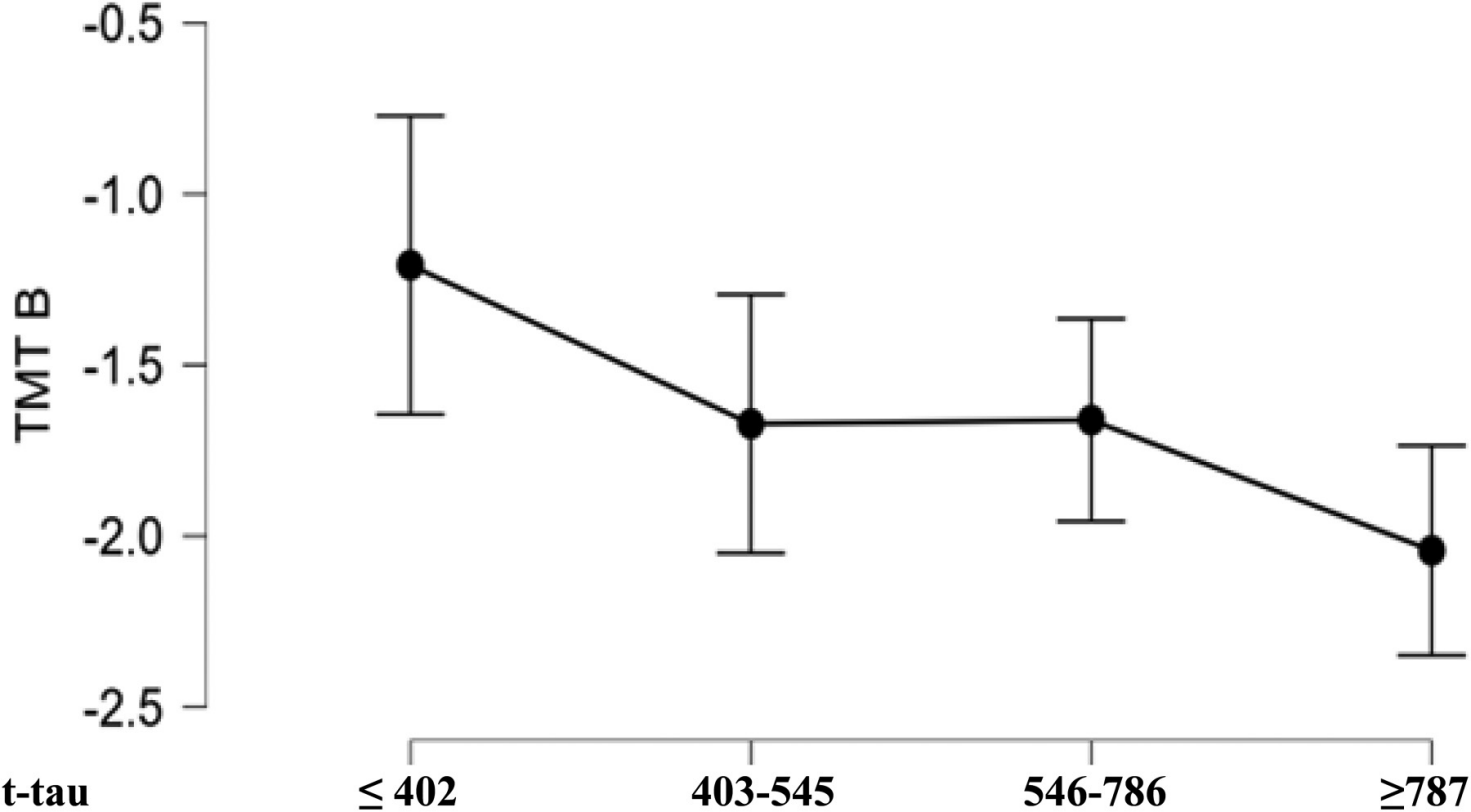
Trail Making Test-B performance of β-ratio + patients.

The association between TMT-B and t-tau was found to be non-linear. Patients in the highest t-tau range (fourth quartile) showed markedly worse performance compared to those with low t-tau scores (first quartile) (Figure 1). Both medial groups (second and third quartiles) did not differ significantly from each other and the two other groups. This pattern suggests a threshold effect, where TMT-B impairment may become apparent only once tau pathology reaches a higher severity level and extends into frontoparietal association cortex.

To test the specificity of this relationship, additional analyses were conducted to exclude alternative explanations: (1) that it might be driven by global disease processing, as determined by the MMSE status, (2) that the significant difference in the Aβ-ratio (Table 1) explains the relationship, and (3) that the association could reflect unspecific vascular or neuroinflammatory processes (i.e., determined by the Albumin ratio).

Firstly, there was no significant difference in MMSE scores between the four t-tau quartiles, and no association between MMSE scores and t-tau was found in the Aβ-ratio + group. The difference in MMSE scores between the lowest and highest t-tau quartiles was only 1 point (Table 1), suggesting that t-tau level is not related to current global cognitive status. Decline in memory performance is the primary clinical symptom used to diagnose AD, and MMSE scores primarily distinguish between patients with normal and severely impaired memory^30^ irrespective of how quickly this state was reached or progresses. Table 1 also shows that age had no impact on the t-tau level, which is of interest even if the TMT-B scores were corrected for age, gender and education, because tau levels are associated with aging.

Secondly, the Aβ-ratio independently predicted t-tau levels, with decreases in Aβ-ratio occurring only in the highest t-tau quartile (Table 1). This indicates that the strongest amyloid pathology was present in individuals with the highest tau levels. However, this pattern did not account for the association between t-tau and TMT-B performance, suggesting that the TMT-B/t-tau relationship is not driven by differences in amyloid pathology.

**Figure 2.**
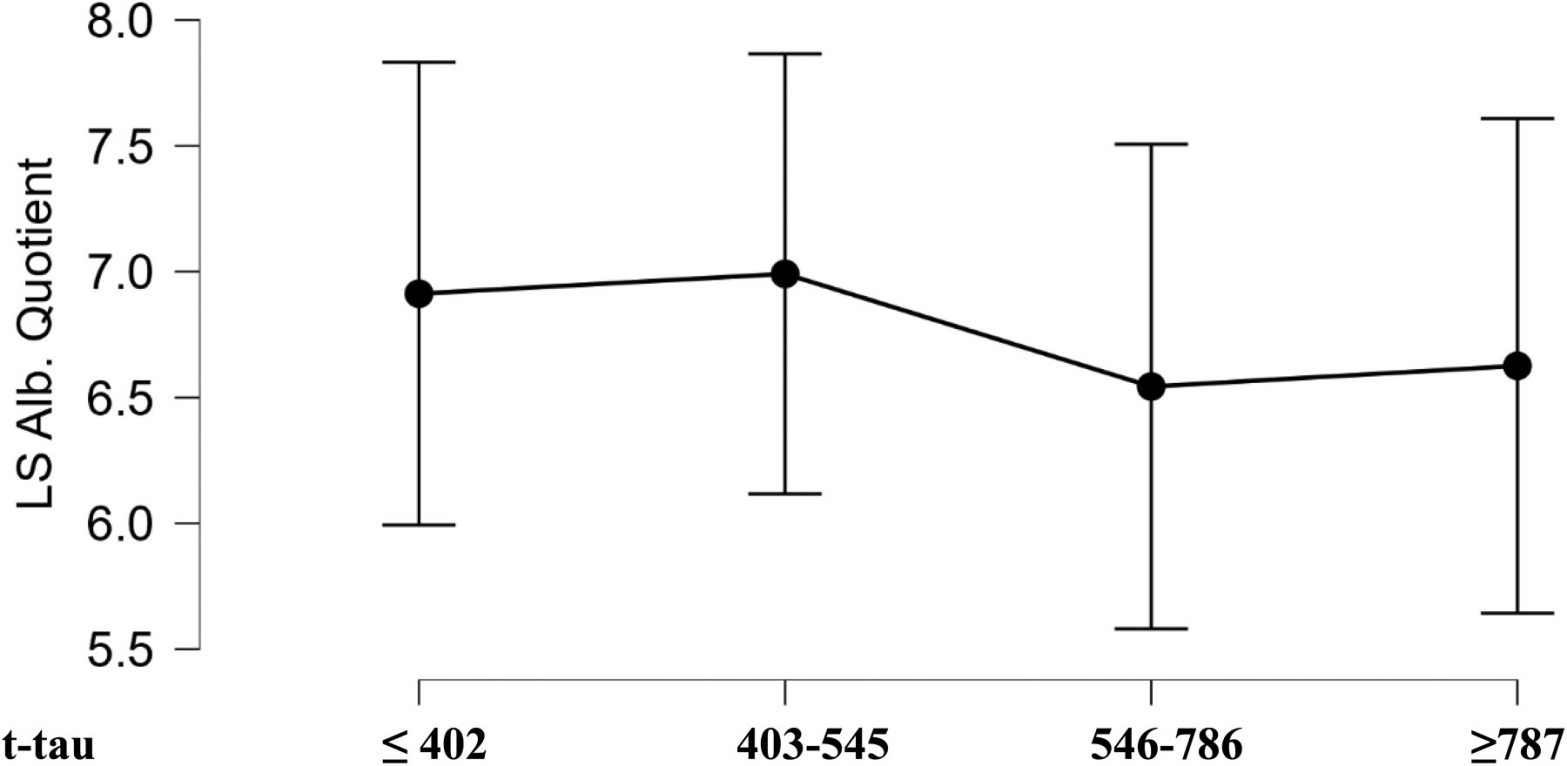
Liquor/serum albumin quotient of Aβ-ratio + patients.

Thirdly, although BBB permeability has been known to moderate the relationship between white matter lesions, cognitive performance, and t-tau in early onset AD,^31^ the albumin quotient did not affect the relationship between TMT-B and t-tau in the present Aβ+ sample (Figure 2).

Taken together, these findings suggest that TMT-B performance reflects both the degree and the cortical spread of tau-related neurodegeneration in Aβ-ratio + patients, independent of global cognitive decline or vascular factors. This highlights TMT-B as a promising clinical marker of disease activity and a potential indicator of progression in AD. In clinical practice, TMT-B is already widely established as part of standard diagnostic assessments in AD. However, its interpretation may evolve in light of these findings. If replicated in longitudinal studies, TMT-B performance may not only reflect executive dysfunction but also provide clinically relevant information on disease activity. It may indicate a more advanced stage of neurodegeneration, thereby supporting patient counseling and treatment planning. In this context, TMT-B findings may help contextualize biomarker results when discussing disease stage, prognosis, and the potential benefit of stage-dependent therapeutic options.

### Limitations

Our study has several limitations. First, the number of patients per t-tau quartile group was approximately 40, representing a relatively small group size. Nevertheless, the central association between t-tau and TMT-B performance was confirmed in the regression analysis, which encompassed more than 140 patients overall.

Second, the current analyses provide only limited insight into a potential vascular component in the association between t-tau and TMT-B, as it was only controlled for the albumin quotient. However, previous studies found contradictory results regarding whether vascular lesions increase AD neurodegenerative processes.^32–34^ Therefore, the impact seems to be limited.

Third, the explained variance of t-tau by TMT-B in the initial regression analysis was significant but low. It should be noted that splitting the group into t-tau quartile subgroups suggests that the association may not be linear. A non-linear fitting might increase the explained variance.

Fourth, the prognostic value of t-tau and TMT-B performance for disease progression can only be demonstrated in longitudinal studies. The present findings primarily offer a rationale for future research, highlighting TMT-B as a promising neuropsychological test to be included in longitudinal designs.

### Conclusions

Clinically, diagnostic findings should be complemented by the best available evidence on the likely course of the disease. The combination of low t-tau and only mildly impaired TMT-B performance may indicate a better prognosis compared to high t-tau and impaired TMT-B. Such information may assist clinicians in prioritizing emerging AD treatments and in estimating the time frame within which independent living can be maintained. As of now, this preliminary evidence requires confirmation through longitudinal studies to determine the true prognostic value of t-tau and TMT-B performance over time.
